# Exploring the association of hyperhomocysteinemia with early pregnancy losses: A retrospective case–control study in a tertiary clinic in Türkiye

**DOI:** 10.1097/MD.0000000000039101

**Published:** 2024-08-09

**Authors:** Gul Ozel Dogan, Orhan Sahin

**Affiliations:** a Department of Gynecology and Obstetrics, University of Health Sciences, Sisli Hamidiye Etfal Education and Research Hospital, Istanbul, Türkiye.

**Keywords:** folic acid, homocysteine, hyperhomocysteinemia, micronutrient deficiency, pregnancy, pregnancy outcomes

## Abstract

A disturbance in the metabolism of homocysteine in both the mother and the fetus has been implicated in several placental vasculopathy-related disorders, including pregnancy loss. This study aimed to provide insights into the potential role of homocysteine, Vitamin B12, and folic acid in early pregnancy losses, with a specific focus on the Turkish population. The results of 93 pregnant women who experienced miscarriage between 5 and 14 gestational weeks and 93 healthy pregnant women at the same gestational weeks were compared. The demographic and pregnancy characteristics of all pregnant women were recorded. Vitamin B12, folic acid, and homocysteine levels were measured in serum samples obtained from the groups at similar gestational weeks. In addition, any associations between these biomarkers and different types of pregnancy loss, such as spontaneous abortion and missed abortion, were evaluated. Vitamin B12 and folic acid serum levels were significantly lower in women with miscarriages (*P* = .019, *P* < .001, respectively). Homocysteine levels were higher in the patient group (*P* < .001). Logistic regression analysis showed that a higher homocysteine level was the only predictive factor of miscarriage (*P* = .001, odds ratio = 0.596); however, folic acid and Vitamin B12 were not predictive factors. There was no significant difference in homocysteine and micronutrient levels between women with missed abortions and women with spontaneous abortions (*P* > .05). Our results support the continuing evidence of a link between maternal homocysteine levels and fetal loss. However, in exploring the shared pathways in the underlying mechanisms causing the 2 forms of pregnancy loss, maternal blood analysis showed no relationship.

## 1. Introduction

Pregnancy loss, particularly miscarriage, or abortus, poses a significant challenge in obstetrics and gynecology, affecting millions of women worldwide. Despite advancements in prenatal care, the underlying mechanisms contributing to pregnancy loss remain incompletely understood.^[1,2]^ Homocysteine, a sulfur-containing amino acid, plays a crucial role in various metabolic pathways, including methionine metabolism. Hyperhomocysteinemia, characterized by elevated blood levels of total homocysteine, has emerged as a potential factor implicated in adverse pregnancy outcomes, including miscarriage.^[3]^ Therefore, the viability of pregnancy may be threatened by elevated homocysteine levels, or hyperhomocysteinemia, which have been linked to thrombosis, oxidative stress, and endothelial dysfunction.^[3–5]^

Pregnancy’s first trimester is a crucial time characterized by significant physiological changes and rapid fetal development. Earlier research has looked into the connection between hyperhomocysteinemia and unfavorable pregnancy outcomes, like miscarriage, during this critical period.^[6,7]^ However, limited research has focused specifically on the Turkish population, where cultural, genetic, and environmental factors may influence the prevalence and impact of hyperhomocysteinemia on pregnancy outcomes.^[8,9]^

It is vital to understand the relationship between hyperhomocysteinemia and first trimester pregnancy losses for several reasons. First, finding biomarkers that anticipate pregnancy problems can help with early management and risk stratification, which may lessen the likelihood of unfavorable outcomes. Equally important, determining how hyperhomocysteinemia contributes to pregnancy loss could lead to the discovery of new treatment targets that could be used to reduce these risks.

The purpose of this study is to investigate if hyperhomocysteinemia is a useful biomarker for predicting pregnancy losses in Turkish women in the first trimester. By examining blood levels of homocysteine, Vitamin B12, and folic acid in both healthy pregnant individuals and those with a history of abortus, we aimed to explore the association between the metabolism of methionine and pregnancy outcomes in the Turkish population. Furthermore, we investigated whether hyperhomocysteinemia could serve as a reliable biomarker for the prompt detection and subsequent management of missed abortus, thereby enhancing prenatal care and lessening the psychological and physical effects of pregnancy loss on those who experience it.

## 2. Materials and methods

This prospective case–control study was approved by the Ethics Committee of the University of Health Sciences, Sisli Etfal Training and Research Hospital, Istanbul, Türkiye (Study Protocol Number: 2024-4363), and was performed in accordance with the Declaration of Helsinki. Written informed consent was obtained from all participants. Women with pregnancies who were admitted to the obstetric clinic for 12 months, from March 1, 2023, to March 1, 2024, were enrolled in the study. The demographic features, chronic diseases, and blood parameters of women with pregnancy loss and healthy pregnant women were recorded.

### 2.1. Participants

Ninety-three pregnant women with miscarriage between 5 and 14 gestational weeks, aged over 18 years, and 93 healthy pregnant individuals who were perfectly matched with this group in terms of age, gestational week, and parity were enrolled in the study. All participants were between the ages of 18 and 40, had a singleton pregnancy, and did not receive any prophylactic nutrition supplements at their admission. Patients’ data were recorded in a database that included information about their clinical and demographic features and serum levels of Vitamin B12, folic acid, and homocysteine. Both groups were questioned regarding demographic characteristics such as age, smoking, alcohol consumption, consanguineous marriage, and chronic diseases, which were recorded in the case forms.

None of the participants had any anatomic or genetic abnormalities, endocrine disorders, chronic inflammatory diseases, or maternal–fetal blood group incompatibility. Pregnant women under the age of 18 and over the age of 40, women with autoimmune diseases and chronic diseases related to high blood pressure, patients with multiple pregnancies, and women with pregnancy loss outside of 5 to 14 gestational weeks or with a positive fetal heartbeat were excluded.

The gestational week of these patients was calculated and recorded according to the last menstrual date. Those included in the pregnancy loss group were divided into 2 subgroups: spontaneous abortion (sudden abortion) and missed abortion. The spontaneous abortion group consisted of patients who presented with bleeding and were diagnosed with a complete or incomplete abortion. The missed abortion group consisted of anembryonic pregnancies in which the gestational sac was larger than 25 mm and no fetal image was observed, and/or patients with a fetal image larger than 7 mm without fetal heart activity. Every patient had an ultrasonographic examination using an ACUSON X300 model device by SIEMENS, conducted by the same doctor.

### 2.2. Collection of samples

After 8 hours of fasting, morning venous blood samples were collected into vacuum gel tubes to investigate hemogram, International Normalized Ratio (INR), among all participants. Total plasma homocysteine was measured using a commercially available enzymatic cycling assay (Cobas 8000; Roche). The reference range was taken as 5 to 15 μmol/L. Vitamin B12 levels in the samples were measured by chemiluminescence emission technique in ROCHE COBAS 8000. The reference range was 197 to 771 ng/L. Folic acid levels were measured by chemiluminescence emission technique in ROCHE COBAS 8000. The reference range was taken as 3.89 to 26.8 μg/L.

### 2.3. Statistical analysis

IBM SPSS Statistics for Windows, Version 21.0 was utilized for the analysis of descriptive and bivariate statistics, numerical outcome predictions, and predictions for identifying groups. Descriptive statistics were given as numbers and percentages for categorical variables and as means, standard deviations, minimums, maximums, and medians for numerical variables. In 2 independent groups, numerical variables were analyzed by the Mann–Whitney *U* test because the normal distribution condition was not met. Proportions in the groups were compared with the Chi-Square test. Logistic regression analysis was carried out to determine significant predictors of miscarriage prior to 14 weeks’ gestation.

We utilized restricted cubic spline (RCS) logistic regression analysis to model the nonlinear relationships between predictor variables and the outcome of interest. Additionally, for further statistical analysis of the current study, R, an open-source software environment for statistical computing and graphics developed by the R Foundation for Statistical Computing (version 4.0.2, released in 2020), was utilized. Furthermore, to evaluate the performance of the logistic regression model, we generated receiver operating characteristic (ROC) curves and calculated the area under the curve (AUC) using the pROC package in R (version 4.0.2, R Foundation for Statistical Computing, 2020). The statistical alpha significance value was accepted as *P* < .05.

## 3. Results

The ages of the participants ranged between 18 and 37 years. The mean age of women with miscarriage was 31.4 ± 5.66 years, and the mean age of healthy pregnant women was 31.9 ± 6.24 years. There was no statistically significant difference between the age distributions of the patients who participated in the study (*P* > .05). The mean gravida, parity, mean gestational week according to the last menstrual period, and miscarriage frequencies were significantly higher in the women with miscarriage group (*P* < .001, *P* = .014, *P* < .001, *P* < .01, respectively). The 2 groups did not differ statistically significantly in terms of smoking, consanguineous marriage, or Rh incompatibility (*P* > .05). None of the pregnant women who participated in our study used alcohol or had chronic diseases. Table 1 shows the demographic and clinical characteristics of the participants.

**Table 1:** The demographic and clinical characteristics of participants.

Characteristics		Women with healthy pregnancy (Mean ± SD)	Women with miscarriage (Mean ± SD)	*P* value
Age		31.9 ± 6.24	31.4 ± 5.66	.61*
Gravida		2.67 ± 1.66	1.97 ± 1.09	<.001*
Parity		1.10 ± 1.05	0.742 ± 0.896)	.014*
Abortion		0.462 ± 0.973	0.140 ± 0.457	<.01*
Last menstrual period		8.12 ± 2.40	11.8 ± 1.09	<.001*
Smoking (n)		2 (2.2%)	0	.597†
Alcohol (n)		0	0	‐
Consanguineous marriage (n)		5 (5.4 %)	0	.059†
Chronic disease (n)		0	0	
RH incompatibility (n)		16 (17.0 %)	14 (15%)	.691‡
Diagnosis	Missed abortion (n)	73 (78.4 %)	‐	‐
	Spontaneous abortion (n)	20 (21.6 %)	‐	‐

n = number of patients.

* Welch test.

† Fisher Exact test.

‡ Chi-Square test.

Vitamin B12, folic acid, and homocysteine serum levels were found to be significantly lower in women in the miscarriage group than in the control group (*P* = .021, *P* < .001, *P* < .001, retrospectively). However, serum hemoglobin and INR levels did not show a statistically significant difference among both groups (*P* > .05). Table 2 demonstrates the comparison of maternal blood analysis results among participants.

**Table 2:** The comparison of maternal blood analysis results of participants.

First trimester maternal blood analysis	Women with miscarriage Mean ± SD	Women with healthy pregnancy Mean ± SD	*P* value
Hemoglobin	11.8 ± 1.12	11.9 ± 0.869	.420*
INR	1.03 ± 0.0948	1.02 ± 0.066	.573*
Vitamin B12	276 ± 135	320 ± 127	.021*
Folic acid	10.1 ± 85.13	14.4 ± 5.45	<.001*
Homocysteine	9.54 ± 4.28	6.24 ± 1.46	<.001*

INR = International Normalised Ratio.

* Welch test.

**Figure 1.**
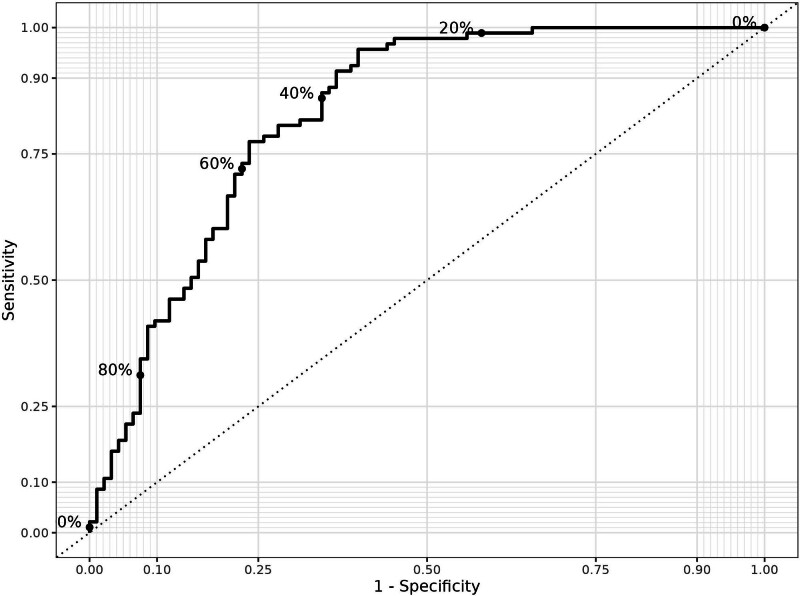
Reviewer operating characteristic (ROC) curve of homocysteine for prediction of miscarriage (the AUC = area under the curve value for homocysteine: 0.8271).

Logistic regression analysis showed that high homocysteine levels were found to be predictive factor for miscarriage (*P* = .001, OR = 0.596); however, miscarriage was not statistically related to low Vitamin B12 and folic acid levels, gravida, and parity (*P* > .05). A multivariable linear regression model in first trimester maternal blood analysis is demonstrated in Table 3. The ROC curve was generated to evaluate the performance of the multivariate logistic regression model, including predictors: Vitamin B12, folic acid, homocysteine, gravida, and parity. The AUC was 0.8271, indicating good model discrimination (Fig. 1).

**Table 3:** Multivariable linear regression model for the first trimester maternal blood analysis.

First trimester maternal blood analysis	*P* value	Odds ratio (OR)	95% CI	
Vitamin B12	.593	1.001	0.998	1.003
Folic acid	.230	1.043	0.974	1.118
Homocysteine	.001	0.596	0.488	0.726
Gravida	.077	0.652	0.411	1.004
Parity	.950	1.020	0.558	1.887

**Figure 2.**
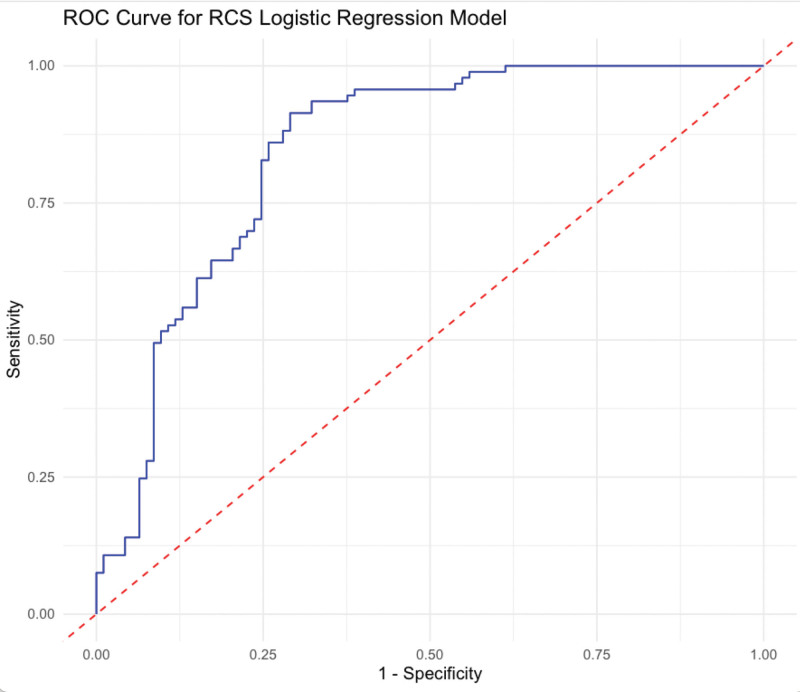
Reviewer operating characteristic (ROC) curve of homocysteine for restricted cubic spline (RCS) logistic regression model on miscarriage (the AUC = area under the curve value for homocysteine: 0.8432).

To better understand the nonlinear relationships between our variables and the outcome, we applied a RCS logistic regression model (Fig. 2). This method allowed us to model the potential nonlinear effects of Vitamin B12, folic acid, and homocysteine on the outcome. The results from the RCS model are summarized in Table 4. Homocysteine showed a significant negative effect on the outcome with an odds ratio of 0.22 (95% CI: 0.07–0.67, *P* = .0012). The model’s discrimination ability was good, with an AUC of 0.8432, as depicted in the ROC curve. These findings show that higher levels of homocysteine were associated with a decreased likelihood of miscarriage, while the effects of Vitamin B12, folic acid, gravida, and parity were not statistically significant.

**Table 4:** Results of restricted cubic spline (RCS) model.

First trimester maternal blood analysis	*P* value	Odds ratio (OR)	95% CI	
Vitamin B12	.394	1.589	0.6403	3.9472
Folic acid	.278	1.775	0.6325	4.9850
Homocysteine	.001	0.222	0.0734	0.6724
Gravida	.074	0.419	0.1621	1.0874
Parity	.899	0.921	0.2608	3.2554

The women with miscarriage group were divided into 2 subgroups: patients with missed abortions and patients with spontaneous abortions. Demographic, clinical, and blood analysis results were compared. We did not find any statistically meaningful differences in terms of age, gravida, parity, history of abortion, gestational week according to last menstrual period, smoking, consanguineous marriage, or RH incompatibility in subgroups (*P* > .05). In addition, serum hemoglobin, INR, Vitamin B12, folic acid, and homocysteine levels were not statistically significantly different (*P* > .05). Tables 5 and 6 show the comparison of the demographical, clinical, and blood analysis results of patients with missed abortions and patients with spontaneous abortions.

**Table 5:** The comparison of demographic and clinical caharacteristics of women with miscarriage subgroups.

Characteristics	Women with missed abortion Mean ± SD (n = 73)	Women with spontaneous abortion Mean ± SD (n = 20)	*P* value
Abortion, median	0.5 ± 0.8 0–3 (0)	0.4 ± 1.5 0–6 (0)	.533*
Age, mean** ± **SD	31.8** ± **6.04	32.0** ± **7.10	.890*
Alcohol (n)	0	0	‐
Smoking (n)	2 (2.9%)	0	1.000†
Chronic disease (n)	0	0	
Consanguineous marriage (n)	3	2	.290†
Gravida, median	2.00 [1.00; 4.00]	2.50 [1.00; 3.25]	.970*
Last menstrual period, median	7.00 [6.00; 9.00]	7.00 [6.00; 8.25]	.570*
Parity, median	1.00 [0; 2.00]	1.00 [0; 2.00]	.860*
RH incompatibility (n)	15	1	.179†

n = number of patients, SD = standard deviation.

* Mann–Whitney *U* test.

† Fisher Exact test.

**Table 6:** The comparison of maternal blood analysis results of women with miscarriage subgroups.

First trimester maternal blood analysis	Women with missed abortion Mean ± SD Min–Max (n = 73)	Women with spontaneous abortion Mean ± SD Min–Max (n = 20)	*P* value
Hemoglobin	11.9 ± 1.2	11.4 ± 2.69	.140*
INR	1.01 ± 0.04	1.0 ± 0.20	.290*
Vitamin B12	251 ± 125	261 ± 137	.970*
Folic acid	10.9 ± 5.22	10.4 ± 4.90	.710*
Homocysteine	8.40 ± 4.27	9.50 ± 4.46	.200*

n = number of patients, INR = International Normalised Ratio.

* Mann–Whitney *U* test.

## 4. Discussion

Hyperhomocysteinemia has been linked to various vascular diseases, raising questions about its possible impact on pregnancy outcomes. The purpose of this study was to assess this connection by exploring the levels of serum homocysteine, folic acid, and Vitamin B12 in women experiencing miscarriage during the first trimester, comparing them to women with healthy and uncomplicated pregnancies. Moreover, we aimed to assess any associations between these biomarkers and different types of pregnancy loss, such as spontaneous abortion and missed abortion. Consistent with existing literature, the results of the current study indicate a significant relationship between hyperhomocysteinemia and miscarriage. In this study, women with miscarriages had notably elevated levels of homocysteine compared to those with healthy pregnancies. Furthermore, our study showed lower levels of folic acid and Vitamin B12 in women with miscarriage, underscoring the potential importance of these micronutrients in maintaining pregnancy viability. Our advanced statistical analyses further revealed that homocysteine levels might serve as predictive factors for pregnancy loss. The importance of these findings is particularly meaningful in the context of our study population, which focused on Turkish participants. This study highlights the relevance of considering regional variations in the evaluation of pregnancy losses. Interestingly, our comparison between spontaneous abortion and missed abortion did not reveal significant differences in serum biomarker levels. This finding suggests that these 2 forms of pregnancy loss may share similar pathways in the underlying mechanisms related to homocysteine metabolism and micronutrient deficiencies.

When the essential amino acid methionine is metabolized, 2 main metabolic pathways result in the formation of homocysteine, an intermediate metabolite. These pathways are trans-sulfuration, where Vitamin B6 functions as an enzyme co-factor, and remethylation, which requires sufficient serum folate and Vitamin B12 as enzyme co-factor.^[10]^ Hyperhomocysteinaemia has been associated with vascular disease, although its potential role in pregnancy outcomes is yet uncertain. The levels of homocysteine decrease during a healthy pregnancy.^[11]^ A disturbance in the metabolism of homocysteine in both the mother and the fetus has been linked to a number of placental vasculopathy-related disorders, including abruption and preeclampsia, as well as recurrent pregnancy loss. In addition, miscarriage is still a unique condition of human pregnancy with a great impact on maternal morbidity and mortality worldwide, especially in developing countries.^[12]^ The etiology is still unknown, and the pathophysiology of it is the subject of detailed investigation to prevent the repetition of pregnancy loss. This study aimed to provide insights into the potential role of hyperhomocysteinemia in predicting and preventing pregnancy losses, with a specific focus on the Turkish population. The results of higher plasma homocysteine levels in women with miscarriage compared to women with healthy pregnancies support a link between altered homocysteine metabolism and adverse pregnancy outcomes, including pregnancy loss. However, interestingly, we found that the levels of homocysteine were not statistically significant in the miscarriage subgroups. Upon review of the literature, it appears that there is limited data on this topic. We believe that the results of our research will open up new avenues for future research in this area.

Homocysteine levels in the Turkish population in the age group 21 to 70 were detected at around 12.81 µmol/L in females.^[13]^ This result is found to be significantly higher than that of healthy individuals from Thailand,^[14]^ Chili,^[15]^ Finland,^[16]^ and Saudi Arabia.^[17]^ Under normal conditions, homocysteine levels reduce during pregnancy.^[18]^ Even though the mean homocysteine levels in women with miscarriages and those with healthy pregnancies in this study were observed to be lower than the general healthy Turkish female population, a meaningful decrease was detected specifically in women with miscarriage, suggesting a probable relationship between elevated homocysteine levels and miscarriage. Therefore, our findings may be considered to represent as an important population-based reference value.

In a study by Ubeda et al, the authors investigated the relationship between pregnancy-induced changes in total homocysteine and folate and Vitamin B12 nutritional status, genetic polymorphism in the methylenetetrahydrofolate reductase enzyme, and gestation outcome.^[19]^ They reported that total homocysteine concentration was not significantly influenced by maternal genotype. Their results indicated that folate was the single negative predictor of maternal homocysteinemia in the first trimester of pregnancy. The current study focuses on the effects of plasma homocysteine, B12 Vitamin, and folate levels on miscarriage. Consistent with their results, the current study also reveals decreased plasma levels of Vitamin B12 and folate in women with miscarriage, thereby reinforcing their conclusion regarding the impact of altered nutritional status on gestational outcomes.

Numerous factors, such as a lack of folic acid and Vitamin B12 deficiency, cause hyperhomocysteinemia. As folate is directly involved in the biosynthesis of DNA, RNA, and proteins, it is essential for the developing fetus. Defective folate metabolism, including hyperhomocysteinemia and Vitamin B12 insufficiency, is known to raise the risk of neural tube defects, fetal abnormalities, placental abruption, and heart defects.^[20]^ First trimester plasma folate deficiency affects 0.5% and Vitamin B12 deficiency affects 29.8% of the Turkish population, in which mandatory micronutrient fortification is absent^.[21]^ Research to date has shown a significant association between maternal serum folic acid, and Vitamin B12 status, and the presence of miscarriage^.[22,23]^ In a study from our country, it was found that folate and vitamin B12 deficiencies were relatively common in the pregnant population.^[24]^ In a cohort study conducted in the perinatology unit in eastern Türkiye, it was reported that low folate status coupled with Vitamin B12 deficiency is strongly associated with neural tube defects. Pregnant women in low- and middle-income countries may benefit from taking folic acid supplements, according to a recently published Cochrane review.^[25]^ Our findings support the fact that, in situations where women’s diets are primarily composed of vegetables, Vitamin B12 supplementation in addition to folate may be taken into consideration for the wellbeing of the fetus.

The Turkish Ministry of Health advises women without a diagnosed chronic condition to consume 400 mg of folic acid supplements daily during the initial 3 months, beginning 1 to 3 months prior to planned conception.^[26]^ The participants in this research did not have any supplements before their administration to our clinic. Due to the exclusion of individuals taking folic acid and Vitamin B12 supplements, this could potentially yield more realistic results, as we observed differing plasma values of homocysteine during pregnancy and its importance for pregnancy losses in a Turkish population. Our advanced statistical analyses further revealed that homocysteine levels might serve as predictive factors for pregnancy loss. This underscores the potential clinical utility of monitoring this biomarker in early pregnancy to identify individuals at risk and implement appropriate interventions.

Our research has several limitations. Firstly, it is based on data gathered from a hospital-based, tertiary clinic. Additionally, the women involved in the study were not monitored throughout their pregnancies, preventing an assessment of their ongoing micronutrient status. Despite these limitations, the study’s strength lies in its relatively large sample size, consisting of participants not taking any supplements and sharing similar demographic and clinical characteristics. Moreover, our study highlighted the need for further research to address and evaluate various issues concerning micronutrient supplementation during pregnancy, and it detected the specific etiological factors contributing to each type of miscarriage to explore potential differences in our country.

In conclusion, our study adds to the growing body of evidence demonstrating the significance of hyperhomocysteinemia, along with deficiencies in folic acid and Vitamin B12, as potential predictors of early pregnancy loss. In order to improve pregnancy outcomes, these findings highlight the significance of early screening and intervention techniques targeted at optimizing maternal micronutrient status. Future research should focus on elucidating the underlying mechanisms linking hyperhomocysteinemia to adverse pregnancy outcomes.

## Acknowledgments

We would like to thank the participants and our department’s staff for their contributions to this study.

## Author contributions

**Conceptualization:** Gul Ozel Dogan, Orhan Sahin.

**Data curation:** Gul Ozel Dogan.

**Investigation:** Gul Ozel Dogan.

**Methodology:** Gul Ozel Dogan, Orhan Sahin.

**Project administration:** Gul Ozel Dogan.

**Resources:** Gul Ozel Dogan.

**Supervision:** Gul Ozel Dogan, Orhan Sahin.

**Writing – original draft:** Gul Ozel Dogan.

**Writing – review & editing:** Gul Ozel Dogan, Orhan Sahin.
